# Editorial: Stem cell therapy in dentistry and oral and maxillofacial abnormalities

**DOI:** 10.3389/fcell.2024.1480398

**Published:** 2024-09-06

**Authors:** Ali Golchin, Ilya D. Klabukov, Yaşar Murat Elçin, Arash Khojasteh

**Affiliations:** ^1^ Department of Applied Cell Sciences, Urmia University of Medical Sciences, Urmia, Iran; ^2^ Department of Regenerative Medicine, National Medical Research Radiological Center of the Ministry of Health of the Russian Federation, Obninsk, Russia; ^3^ Tissue Engineering, Biomaterials and Nanobiotechnology Laboratory, Ankara University Faculty of Science, and Ankara University Stem Cell Institute, Ankara, Türkiye; ^4^ Department of Oral and Maxillofacial Surgery, School of Dentistry, Shahid Beheshti University of Medical Sciences, Tehran, Iran

**Keywords:** stem cells, stem cell therapy, cell-based therapy, regenerative medicine, dentistry, maxillofacial abnormalities

Stem cell research in dentistry has garnered significant interest due to its potential for regenerating damaged dental and maxillofacial tissues. The field of dental and maxillofacial regeneration is experiencing a renaissance, driven by groundbreaking research that is pushing the boundaries of traditional treatments. Currently, cell technologies allow bridging the gap with the potential to revolutionize clinical practices and improve patient outcomes in dentistry, oral and maxillofacial surgery. However, several challenges still need to be addressed. It is crucial to consider the emerging obstacles associated with using stem cells in regenerative dentistry and to evaluate their effectiveness compared to traditional treatments. Over the past few decades, the rise of stem cell research, in conjunction with tissue engineering, has introduced new perspectives in regenerative medicine and, by extension, regenerative dentistry. As researchers look into different stem cell sources, especially those-derived oral tissues, and new ways to make scaffolds, like bio-printing, they are more likely to combine these advances to come up with new approaches to treat patients. However, the majority of emphasis has been directed into therapeutic treatments, while simultaneously, the majority of fundamental questions have been answered. This Research Topic aims to highlight recent research in the field of stem cell applications in dentistry and maxillofacial reconstruction.

The influence of the immune system on tissue repair is still an understudied area of regenerative medicine. Liu et al. performed a review of the interactions between immune cells and stem cells, exploring their interaction and their involvement in jaw development, maintenance of homeostasis, and pathological circumstances. The primary goal of the study was to ultimately explore the relationship between periodontal ligament stem cells, dental pulp mesenchymal stem cells, jawbone mesenchymal stem cells, and Schwann cells in the immune microenvironment of the jaw.

A primary concern in the field of regenerative dentistry is the enhancement of odontogenesis by improving differentiation and proliferation of specific stem cells. In this regard, Inubushi et al. suggested enhancing dentin production in odontoblasts by utilizing a distinctive chlorinated oxidant known as Matching Transformation System^®^ (MA-T). They found that via activating the canonical Wnt signaling pathway, MA-T treatment in odontoblasts decreased the sulfation of HSPG and increased the levels of dentin sialophosphoprotein (Dspp) and Dentin Matrix Protein 1 (Dmp1). Furthermore, the use of MA-T treatments outside of the living organism enhanced the formation of dentin matrix in developing tooth samples. However, a comprehensive investigation is necessary to precisely determine the function of the intricate Wnt signaling network in dentin formation. Moreover, *in vitro* investigation shown that LiCl treatment increased the mRNA expression of Wnt10a and Wnt6 in odontoblasts, but had no effect on Wnt5a. Similarly, MA-T treatment at concentrations of 0.2 ppm and 1.0 ppm also had no effect on Wnt5a expression. The study utilized primary mouse dental papilla mesenchymal cells (mDP cells) to further examine the growth into odontoblasts, chondrocytes, and adipocytes. The study proposes a novel approach to stimulate dentin formation by modifying HSPG. It also elucidates the therapeutic processes of MA-T in promoting the differentiation of odontoblasts Inubushi et al. These findings suggest a promising potential for using pharmaceutical methods to regenerate dental tissues.

In another study, Irfan et al. discovered that disabling the C5a-like receptor 2 (C5L2) CRISPR gene significantly improves the process of mineralization in TNFα-stimulated dental pulp stem cells (DPSCs), and promotes the production of dentin sialophosphoprotein (DSPP) and dentin matrix protein-1. This study has attempted to clarify the function of inflammation in dentinogenesis in order to develop effective ways for engineering stem cell-based approaches Bouland et al.

In a clinical study, Bouland et al. evaluated the capacity of adipose-tissue stromal vascular fraction (AT-SVF) and leukocyte-platelet-rich fibrin (L-PRF) to treat medication-related osteonecrosis of the jaw (MRONJ). As we know, the AT-SVF consists of mesenchymal stromal cells (MSC) and endothelial progenitor cells (EPC) that stimulate the growth of bone tissue. The L-PRF scaffold facilitates tissue repair by releasing growth factors. The study included nine individuals who had a total of ten MRONJ lesions, all of which showed no symptoms of recurrence. Three-dimensional medical imaging demonstrated bone regeneration 6 months post-surgery. The findings indicate a novel therapeutic strategy for MRONJ involving the use of autologous AT-SVF within an L-PRF scaffold Bouland et al.

In addition to teeth and oral bones, other oral related diseases can be targeted for stem cell-based therapies. For instance, the salivary gland hypo-function (SGH) is a medical issue that results in dry mouth, heightened susceptibility to disorders, and diminished quality of life. Present therapies mostly aim to alleviate symptoms and enhance quality of life, but do not restore impaired glands. Pluripotent stem cells (PSCs) are being recognized as a promising treatment option for SGH. Song et al. reviewed the related preclinical studies and demonstrated that PSCs exhibit encouraging immunomodulatory and tissue regeneration properties for SGH. This study provides a comprehensive analysis of the effectiveness and prospective application of PSCs in the treatment of SGH, which can serve as valuable guidance for future research endeavors Song et al.

Overall, there is no doubt that regenerative dentistry will continue to rapidly advance and improve treatment methods as the convergence of disciplines continues, and stem cell-based approaches and tissue engineering continue to improve. These technological breakthroughs show significant potential for the future of dentistry and the treatment of oral and maxillofacial disorders, providing creative ways to progress this discipline.

